# The novel influenza A virus protein PA-X and its naturally deleted variant show different enzymatic properties in comparison to the viral endonuclease PA

**DOI:** 10.1093/nar/gkv926

**Published:** 2015-09-17

**Authors:** Laura Bavagnoli, Stefano Cucuzza, Giulia Campanini, Francesca Rovida, Stefania Paolucci, Fausto Baldanti, Giovanni Maga

**Affiliations:** 1 Institute of Molecular Genetics IGM-CNR, via Abbiategrasso 207, 27100 Pavia, Italy; 2 Molecular Virology Unit, Microbiology and Virology Department, Fondazione IRCCS Policlinico San Matteo, P.le Golgi 2, 27100 Pavia, Italy

## Abstract

The PA protein of Influenza A virus (IAV) encoded by segment 3 acts as a specialized RNA endonuclease in the transcription of the viral genome. The same genomic segment encodes for a second shorter protein, termed PA-X, with the first 191 N-terminal aminoacids (aa) identical to PA, but with a completely different C-ter domain of 61 aa, due to a ribosomal frameshifting. In addition, it has been shown that several IAV isolates encode for a naturally truncated PA-X variant, PAXΔC20, missing the last 20 aa. The biochemical properties of PA-X and PAXΔC20 have been poorly investigated so far. Here, we have carried out an enzymatic characterization of PA-X and its naturally deleted form, in comparison with PA from the human IAV strain A/WSN/33 (H1N1). Our results showed, to the best of our knowledge for the first time, that PA-X possesses an endonucleolytic activity. Both PA and PA-X preferentially cut single stranded RNA regions, but with some differences. In addition, we showed that PAXΔC20 has severely reduced nuclease activity. These results point to a previously undetected role of the last C-ter 20 aa for the catalytic activity of PA-X and support distinct roles for these proteins in the viral life cycle.

## INTRODUCTION

The influenza A virus (IAV) genome consists of eight negative-sense viral RNA (vRNA) segments coding at least for 16 different proteins (1–3). Among these, the PB1, PB2 and PA subunits, encoded by genomic segments 1, 2 and 3, respectively, form together the RNA-dependent RNA polymerase (RdRP) of the virus, an heterotrimer with molecular mass of 250 kDa (4), which is essential for transcriptional and replicative processes. To generate its own mRNAs, the influenza virus requires 5′-capped RNA primers, derived from pre-mRNAs of the host cell through a mechanism called ‘cap-snatching’. The cap-binding domain located in the PB2 polymerase subunit interacts with the 5′-cap structure of a host pre-mRNA, which is then cleaved 10–13 nucleotides (nt) downstream of the cap (5). The viral mRNA is then synthesized using the viral genomic RNA (vRNA) as template by the polymerase activity of the PB1 subunit (6). By structural and mutagenesis studies, it has been shown that the endonuclease activity of the RdRP responsible for the cap-snatching resides in the PA subunit (7,8). Within the active site, the residues Glu80, Asp108, Glu119 and Lys134 along with His41, conserved in all IAVs, coordinate the binding of a divalent cation, essential for the PA endonuclease activity. This activity is highly stimulated by Mn^2+^ and Co^2+^ and less efficiently by Mg^2+^, Ca^2+^, Zn^2+^ and Ni^2+^ (9,10).

Recently, through the analysis of 1300 sequences derived from different isolates, a new highly conserved open reading frame has been discovered, within the same genomic segment coding for the PA subunit, called ORF-X (11). The ORF-X is translated into a new viral protein, called PA-X, thanks to a +1 ribosomal frameshift. The protein PA-X displays an N-terminal (N-ter) region of 191 aminoacids (aa) identical to the one of PA and a completely new C-terminal (C-ter) region consisting of 61 aa.

Since the binding sites of PA for the PB1 subunit are not present in the C-ter region of PA-X, it has been hypothesized that the latter may be an accessory protein acting independently from the RNA polymerase. Cellular studies suggested its involvement in the degradation of host mRNAs, contributing in this way to the modulation of the viral pathogenesis (11,12). To date, PA-X has not been biochemically characterized and the role of its unique C-ter region remains unclear.

PA-X is conserved among influenza viruses (13): it is present in 75% of the known isolates, while the remaining 25% mostly displays the presence of a PA-X protein with a deletion of 20 aa in the C-ter region (PAXΔC20) whose role in viral pathogenesis remains unknown, although it may be an adaptation to increase the viral fitness of the virus into new hosts (14–16).

In this study, by using recombinantly expressed PA, PA-X and PAXΔC20 proteins from the A/WSN/33 (H1N1) human influenza virus, we show, to the best of our knowledge for the first time, that PA-X possesses a robust endonucleolytic activity. Both PA and PA-X preferentially target ssRNA regions, but with some differences, depending on the structure of the RNA to be cleaved. In addition, we characterize for the first time the PAXΔC20 natural variant, showing that it possesses reduced endonucleolytic activity, when compared to PA-X full length. Collectively, our results indicate an important functional role of the last C-ter 20 aa of PA-X and support the idea of distinct roles of PA, PA-X and PAXΔC20 in the viral life cycle.

## MATERIALS AND METHODS

### Chemical reagents

[γ-^32^P] used for RNA labelling was purchased from Hartmann Analytic GmbH (Braunschweig, Germany). The inhibitor of PA endonuclease, 2,4-dioxo-4-phenylbutanoic acid (DPBA), was purchased from MolPort (Riga, Latvia). All other reagents were purchased from VWR International (Radnor, PA, USA) or Sigma-Aldrich (St. Louis, MO, USA).

### Protein expression and purification

The entire PA ORF was amplified from the H1N1 A/WSN/1933 strain and cloned into the pTrcHisA expression vector. This construct was used for site-directed mutagenesis to generate the corresponding expression vectors for PA-X, PA-XΔC20 and PAcd (comprising the first N-ter 191 aa of PA). All proteins were cloned in the bacterial expression vector pTrcHisA and were expressed fused to a N-terminal His_6_-tag. PA-X was generated by the deletion of the cytosine 574 from the PA sequence (counting from the start of the PA ORF), while PAcd and PAXΔC20 were obtained by the insertion of a stop codon after phenylalanine in position 191 and after methionine in position 232, respectively (counting from the start of the PA ORF).

*Escherichia coli* BL21 (DE3) cells transformed with the expression vectors pTrcHisA-PA, pTrcHisA-PA-X, pTrcHisA-PAXΔC20 and pTrcHisA-PAcd were inoculated into 2.5 l of Luria Bertani broth with the addition of ampicillin (50 μg/ml) at 37°C up to OD_600_ of 0.6. The expression of His_6_-PA proteins was induced by adding isopropylthio-D-galactoside (IPTG) to a final concentration of 0.1 mM followed by growth for 16 h at 15°C.

The cells were harvested by centrifugation, resuspended in 1.5 ml lysis buffer (10 mM Tris-HCl pH 8, NP-40, 0.01%, 0.1 M NaPO4 pH 8.0, 5 mM imidazole, 1 mM PMSF, 1X Protease inhibitors) per 1 g of dry pellet, lysed for 30 min on ice by addition of lysozyme to a final concentration of 1 μg/μl and sonicated. The lysed samples were ultra-centrifuged at 26 000 rpm for 1 h at 4°C. His_6_-tagged PA proteins in the supernatant were purified by affinity chromatography through a FPLC-NiNTA column. PA proteins bound to the column were eluted using a linear gradient from 25 mM to 500 mM imidazole pH 8, in 25 mM KPO_4_ pH 7.5, 500 mM NaCl and 10% glycerol.

Dialysis step at 4°C was performed with a slide-A-lyzer 3.500 MWCO dialysis membrane (Pierce), using 20 mM Tris-HCl pH 8, 0.5 mM DTT, 25 mM NaCl, 20% glycerol, as dialysis buffer. The dialysis proceeded for 3 h, changing each hour the dialysis buffer. PA proteins were purified using a MonoQ cation exchanger column (GE Healthcare), with a linear gradient from 0.025 M to 1 M NaCl in 20 mM Tris-HCl pH 8, 0.5 mM DTT, 20% glycerol.

**Figure 1.**
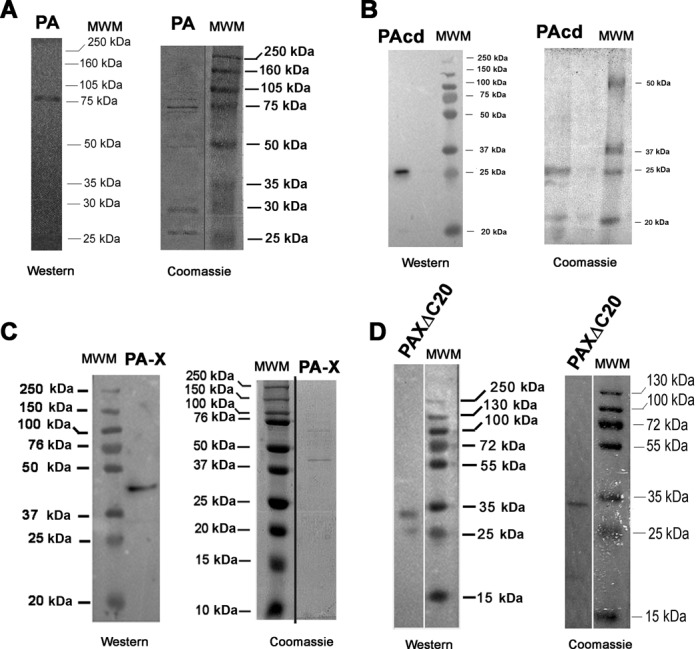
Western blot with anti-his antibodies and Coomassie staining of SDS-PAGE of the final preparations of the recombinant proteins used in this study. (**A**) PA. (**B**) PA catalytic domain. (**C**) PA-X. (**D**) PAXΔC20 mutant.

The protein concentrations were determined by the Bradford assay. A portion of the protein was resolved using SDS-PAGE gel 12% and transferred onto a nitrocellulose membrane. The membrane was blocked in 7.5% (w/v) dried milk dissolved in Tris-buffered saline (TBS) 1X for 2 h at room temperature. The membrane was washed twice with TBST (TBS + Tween 20 0.05%) and once with TBS 1X. For the identification of His_6_-PA proteins, the membrane was incubated O/N at 4°C with primary anti-His_6_ (1:500) rabbit IgG antibody (Cell Signaling), diluited in TBS 1X, 5% w/v BSA, 0.1% Tween-20. Subsequently, HRP-conjugated anti-rabbit secondary antibody (Jackson ImmunoResearch) was added at room temperature for 1h. The development of immunoblots was carried out using chemiluminescence reagents (Pierce Thermo Scientific). Coomassie gels and western blots of the final preparations are shown in Figure 1. Some additional bands can be seen, especially in the PA final preparation. To exclude possible carry-over of a contaminating *E. coli* endonuclease, we also carried out a mock purification through a NiNTA column according to the protocol described above, but using induced *E. coli* cells, transformed with an empty pTrcHisA expression vector. The results are shown in Supplementary Figure S1. No detectable contaminating endonuclease activity was recovered in the fractions eluted by the NiNTA column. This, in addition to the fact that both PA and PA-X nuclease activities were completely abolished by the inhibitor DBPA (Supplementary Figure S3) and that the differences in the catalytic activity between PA and PA-X were substrate-specific (see Results section below), argue against a contribution of these contaminants to the results of the enzymatic tests.

### RNA substrates

The sequences of the RNA oligonucleotides used in this study are given in Table 1.

**Table 1.** Sequences of the RNA oligonucleotides used

RNA substrate	Sequence^a^	Notes
Subst. 1 (38mer)	5′- AGUAGAAACAAGGGUAUUUUUAUACCCUGCUUUUGCUC-3′	from (7)
Subst. 2 (51mer)	5′- GGCCAUCCUGUUUUUUUCCCUUUUUUUUUUUUUUUUUUUUUUUUUUUUUUU-3′	from (10)
Subst. 3 (38mer_10)	5′- AGUAGAAACAAGGGUAUUUUUAUACUGUGCUUUUGCUC-3′	this study
Subst. 4 (26mer)	5′- GCUGUUUUUUUUUUUUCCCAAACAGC-3′	this study
Subst. 5 (U-rich 36mer)	5′- GCUGUUUUUUUUUUUUCCCAAACAGCUUUUUUUUUU-3′	this study
Subst. 6 (r(A)40)	5′- AAAAAAAAAAAAAAAAAAAAAAAAAAAAAAAAAAAAAAAA-3′	this study
18mer_p	5′- CCCAAGAACCCAAGGAAC-3′	this study
18mer_t	5′-GUUCCUUGGGUUCUUGGG-3′	this study; annealed to 18mer_p to generate Substr.7
56mer_t	5′- UUUUUUUUUUACCAGCUUUGUUCCUUGGGUUCUUGGGAGCAGCAGGAAAAAAAAAA-3′	this study; annealed to 18mer_p to generate Substr.8
36mer_t	5′- ACCAGCUUUGUUCCUUGGGUUCUUGGGAGCAGCAGG-3′	this study; annealed to 18mer_p to generate Substr.9
46mer (substrate 10)	5′- AAAAAAAAAAGCUGUUUUUUUUUUUUCCCAAACAGCUUUUUUUUUU-3′	this study
r(U)20 (substrate 11)	5′- UUUUUUUUUUUUUUUUUUUU-3′	this study

^a^The predicted secondary structures adopted by the different RNA sequences have been computed by the web-based tool Mfold (18) and are shown in Scheme 1. According to the prediction, each of the analysed substrates was able to adopt a single secondary structure, which corresponds to the structures shown in Scheme 1. The predicted ΔG values indicated that these secondary structures were stable at 37°C.

All RNA oligonucleotides were purchased by Biomers with a 5′ end fluorescein (FAM) labelling, with exception of the 56mer that bears a 3′ end FAM modification, and of r(A)_40_ and r(U)_20_ which were radioactively labelled with [γ-^32^P] ATP (500 μCurie) at final concentration of 25 μM. The 3′ and 5′ ends of the 38mer oligonucleotide corresponds to the vRNA ends with a short linker (7).

### Annealing reactions

The different RNA oligonucleotides were hybridized to generate the various substrates, as summarized in Table 1. In annealing reactions (50 μl) each RNA substrates was used to a final concentration of 2.5 μM in presence of Annealing buffer (1.5 M KCl, 100 mM Tris-HCl pH 8), and the reactions were incubated in heating block at 90°C for 5 min and slowly cooled O/N.

### Endonuclease assay

**Scheme 1.**
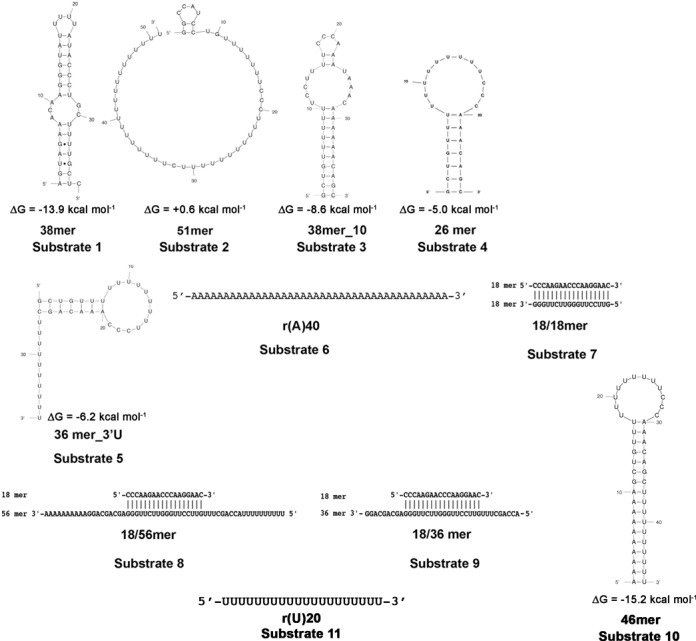
Structures of the substrates used in this study. Secondary structure predictions were made with the web-based tool Mfold (http://mfold.rna.albany.edu/?q=mfold). Only one secondary structure has been predicted by the program to be formed by each substrate and this is shown along with the computed ΔG values.

Endonuclease assays were performed using the RNA substrates reported in Scheme 1. Reactions were set up in a final volume of 10 μl by incubating 2 pmols of each PA proteins (unless otherwise indicated in the figure legends) with 2.5 μM of single strand RNA substrates, and 1.25 μM of double strand RNA substrates. Reaction buffer was 20 mM Tris-HCl pH 8, 100 mM NaCl, 10 mM β-mercaptoethanol, 20 U RNase inhibitor and 1 mM of different cofactors MgCl_2_, MnCl_2_, CoCl_2_, CaCl_2_ (as indicated). Endonuclease reactions were incubated at 37°C for 40 min (unless otherwise indicated in the figure legends) and reactions were stopped by adding 98% deionized formamide, 10 mM EDTA, 0.025% xylene cyanol FF, 0.025% bromophenol blue. Samples were heated at 95°C for 5 min and loaded on 7 M 12% polyacrylamide gel, run at 40 W in TBE 1X. The bands corresponding to degradation products were visualized by laser scanning densitometry on a Typhoon Trio (GE Healthcare) phosphorimager and quantified using the program ImageQuant. Due to the intrinsic sensitivity of RNA to spontaneous hydrolysis and RNase action, some degradation of the RNA substrates in the control reactions was always present, even in the presence of RNase inhibitors. During quantification, we have subtracted this degradation as background, from the bands of interest.

### Kinetic analysis

For the determination of the *K*_m_ and *V*_max_ values, time course experiments were performed in duplicate under the assay conditions described above, in the presence of increasing RNA substrate concentrations. Four different substrate concentrations were used for each set of experiments. Data were subjected to linear interpolation and the slope of each curve was taken as the apparent reaction rate (*k*_app_). Plotting of *k*_app_ values as a function of the substrate concentration was fitted to the Michaelis–Menten equation:

(1) \begin{equation*} k_{\rm app} = {V}_{\max } /(1 + {K}_{\rm m} /{S}) \end{equation*}

Where *V*_max_ is the apparent maximal reaction rate (expressed as pmols x min^−1^x [E]_0_) and *K*_m_ is the apparent Michaelis constant for substrate binding. Each *k*_app_ value was derived from the interpolation of 4 different time points. Data were analysed by the program GraphPad Prism 5.0 using Equation (1) as the fitting algorithm. Standard deviations were calculated by the program during the fitting process. Fitting was performed by the Marquardt-Levenberg least-squares non linear regression method. Representative plots showing the linear interpolation of the pmols of digested substrate and the non linear curve fitting to Equation (1) of the calculated slopes (*k*_app_ values) are shown in Supplementary Figure S2.

## RESULTS

### Cofactor requirements and inhibitor sensitivity of PA and PA-X

**Figure 2.**
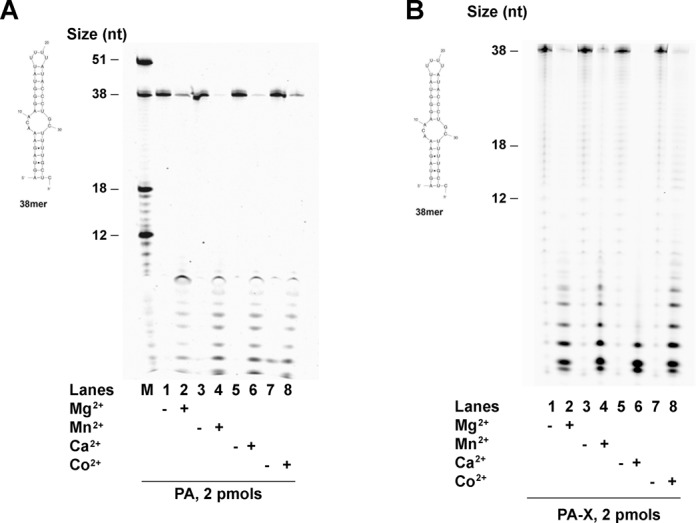
Metal ion activation of the endonuclease activities of PA and PA-X. (**A**) PA was incubated with RNA substrate 1 (Scheme 1) in the absence (lanes 1, 3, 5, 7) or presence (lanes 2, 4, 6, 8) of 1 mM of Mg^2+^, Mn^2+^, Co^2+^ and Ca^2+^. M indicates the molecular marker. The structure of substrate 1 is reported in the left side of each gels. (**B**) As in panel A, but in the presence of PA-X.

Literature data indicated that the endonuclease activity of PA is activated by different metal ions as cofactors (9). As a preliminary test to characterize the activity of our protein preparations, we compared the cofactor requirements of the recombinant PA and PA-X proteins, using substrate **1** (Scheme 1). As shown in Figure 2A, PA was active in the presence of the four different divalent cations Mg^2+^, Mn^2+^, Ca^2+^ and Co^2+^, as already reported (9). Similarly, PA-X showed activity with all four cofactors (Figure 2B). Interestingly, PA-X seemed to be highly stimulated by Ca^2+^, with respect to other metals (Figure 2B, lane 6). The significance of this finding is currently under investigation. To confirm the authenticity of the PA and PA-X proteins, we tested their sensitivity to DBPA, a known inhibitor of the endonuclease activity of PA (17). As shown in Supplementary Figure S3, both PA and PA-X were inhibited by DBPA on substrate **1** (Scheme 1). Together, these results indicated that PA and PA-X share similar cofactor requirements and are both sensitive to DBPA.

### PA cleavage is influenced by the secondary structure of the RNA substrate

**Figure 3.**
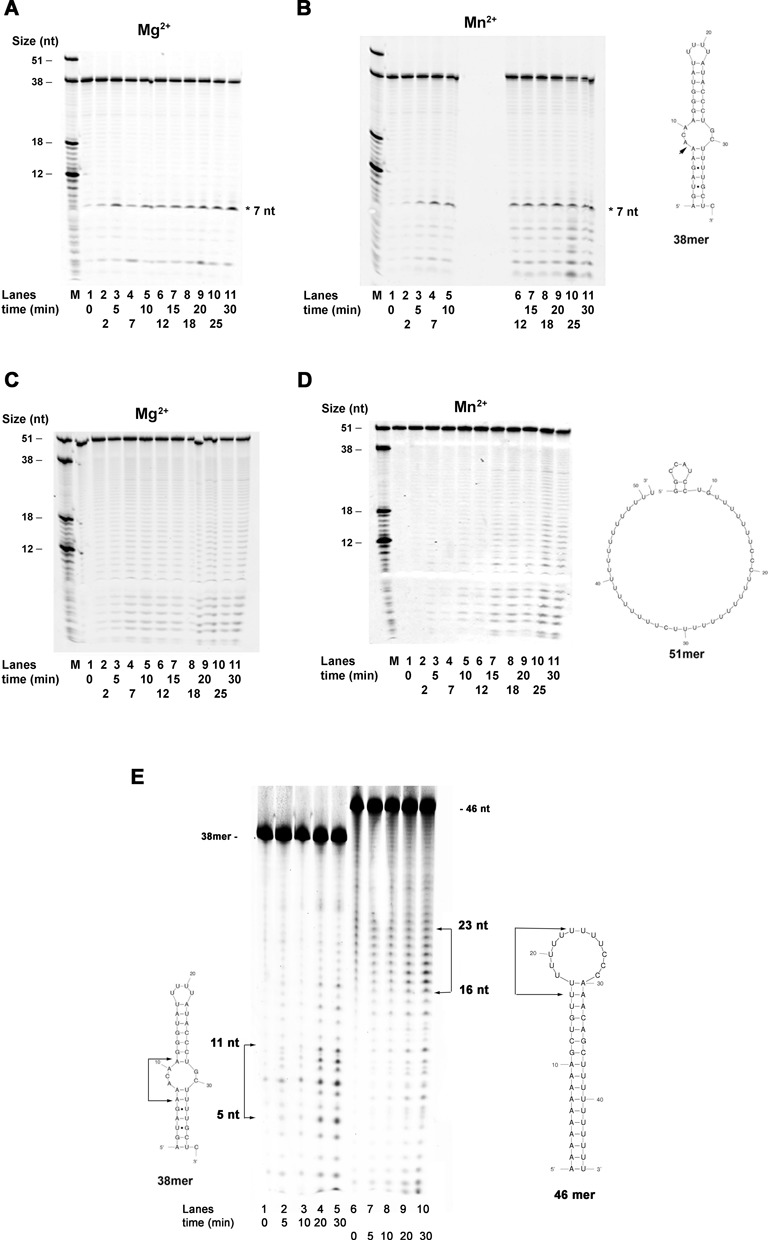
PA endonuclease activity is influenced by the structure of the substrate. The structure of the substrates used is shown next to the panels. 2 pmols of PA were used in all assays. (**A**) Time course assay with PA on substrate 1 (Scheme 1) in the presence of Mg^2+^. M indicates molecular size markers. Lane 1, negative control in the absence of enzyme. (**B**) As in panel A, but in the presence of Mn^2+^. The arrow indicates the position of the +7 product on the substrate sequence. (**C** and **D**) As in panels A and B, but in the presence of substrate 2 (Scheme 1). (**E**) Time course assay with PA on substrate 1 (lanes 1–5) or substrate 10 (lanes 6–10) in the presence of Mg^2+^. Arrows indicate the size of the major cleavage products and their corresponding position on the substrates.

Time course experiments were performed with PA on substrates **1** and **2** (Scheme 1), in the presence of either Mg^2+^ or Mn^2+^ as the metal cofactors. These two oligonucleotides were predicted to form different secondary structures (Scheme 1) (18): substrate 1 can form a stable stem loop with an internal bubble, while substrate 2 was predicted to be mostly in a ss form, with a possible short (8 nt) 5′ stem-loop (the ΔG was however slightly positive, indicating that such loop would be only transiently formed at 37°C). As shown in Figure 3A and B in the presence of both cofactors, on substrate **1** PA generated a main cleavage product at +7, already after 2 min of incubation. This corresponded to cleavage between the two A's at the ss-to-ds junction of the bubble. PA continued the digestion of the dsRNA region at the 5′ end of the substrate, as indicated by the generation of products < 5 nt, especially in the presence of Mn^2+^ (compare lanes 6–11 of Figure 3A with the same of Figure 3B), likely as a result of processive cleavage starting from the adjacent ssRNA region. When tested on substrate **2**, on the other hand, PA displayed a distributive cleavage pattern with the generation of a ladder of products (Figure 3C and D). These results suggest that PA preferentially cleaves ssRNA and it is able to efficiently recognize ss-to-ds RNA junctions within secondary structures. To verify whether ssRNA embedded within dsRNA regions may be a preferential site for PA cleavage, substrate **10** (Scheme 1) was used, which was predicted to form a stable stem-loop with a ssRNA starting at position +17, instead of position +7 as in substrate **1**. As shown in Figure 3E, on substrate **10**, PA generated products starting between 16 nt and 23 nt (lanes 7–10), again consistent with cleavage starting at the 5′ side of the ssRNA loop. PA does not have any known sequence specificity, thus it can in principle cut anywhere on the RNA substrate. The data shown on Figure 3A and E, indicated that PA consistently started to cut within the first nucleotides at the 5′ side of the ssRNA loop, suggesting that the structure (i.e. ssRNA region), rather than sequence of the substrate was recognized as the target site.

### PA-X has endonuclease activity

**Figure 4.**
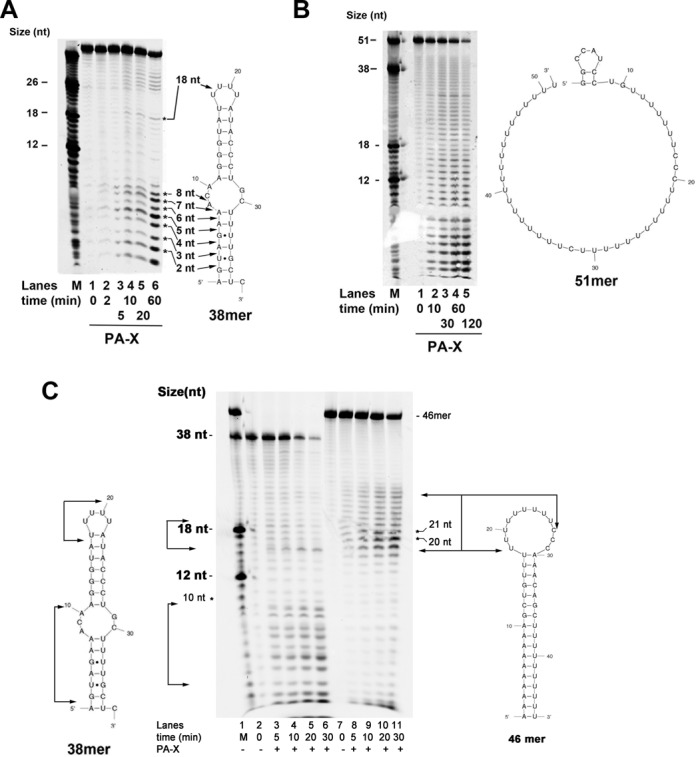
PA-X shows a different cleavage pattern from PA. The structure of the substrates used is shown next to the panels. 2 pmols of PA-X were used on all assays. (**A**) Time course assay with PA-X on substrate 1 (Scheme 1). M indicates the molecular size marker. Lane 1, negative control in the absence of enzyme. The structure of the substrate is shown on the right side of the panel. Asterisks mark the major cleavage products and arrows indicate their corresponding positions on the substrate sequence. (**B**) As in panel A, but on substrate 2 (Scheme 1). M indicates the molecular size marker. Lane 1, negative control in the absence of enzyme. (**C**) Time course assay with PA-X on substrate 1 (lanes 1–6) or substrate 10 (lanes 7–11). Arrows indicate the size of the major cleavage products and their corresponding position on the substrates.

Next, the activity of PA-X was tested on substrates **1** and **2**, in the presence of Mg^2+^. As shown in Figure 4A, on substrate **1** PA-X generated multiple cleavage products. The earliest products (2 min) corresponded to cleavage starting within the internal bubble (+8) down to the first nucleotides at the 5′ end (+2). At later time points (10 min), products corresponding to cleavage within the U-U-U-U loop (position +18) and to the 3′ side of the internal bubble (positions +28 to +31), started to appear. On the other hand, on substrate **2**, PA-X showed a distributive pattern with the generation of a ladder of products similarly to PA (compare Figure 4B with Figure 3C). Quantification of the products as a function of time allowed the calculation of the apparent steady-state rate of digestion for both proteins (*k*_obs_, Table 2). This analysis revealed that PA-X was 2-fold more active than PA on substrate **2**, while both enzymes showed comparable activity on substrate **1**. PA-X was also tested on substrate **10** (Scheme 1). As shown in Figure 4C, when PA-X was incubated with this template, the products that accumulated were almost exclusively those corresponding to cleavage between +17 and +27 positions, corresponding to the ssRNA loop. This indicated that also PA-X recognized as a preferred target site a ssRNA region embedded in a dsRNA structure.

**Table 2.** Steady-state rates for RNA digestion by PA and PA-X proteins

PA	
Substrate	*k*_obs_ (pmols x min^−1^)
1 (38mer)	0.084 ± 0.002
2 (51mer)	0.067 ± 0.002
4 (26mer)	0.06 ± 0.003
5 (36mer_3′U)	0.096 ± 0.006
6 (r(A)40)	0.027 ± 0.002
7 (18/18mer)	0.009 ± 0.002
PA-X	
Substrate	*k*_obs_ (pmols x min^−1^)
1 (38mer)	0.072 ± 0.002
2 (51merU)	0.12 ± 0.003
4 (26mer)	0.036 ± 0.001
5 (36mer_3′U)	0.098 ± 0.004
6 (r(A)40)	0.1 ± 0.004
7 (18/18mer)	0.025 ± 0.008
11 (r(U)20)	0.09 ± 0.008
18mer_p	0.05 ± 0.003

^a^Kinetic parameters and the corresponding ±S.D. have been calculated as detailed in the Materials and Methods section. Values are the mean of two replicates.

### Comparison of PA and PA-X on different structured and unstructured ssRNA substrates

**Figure 5.**
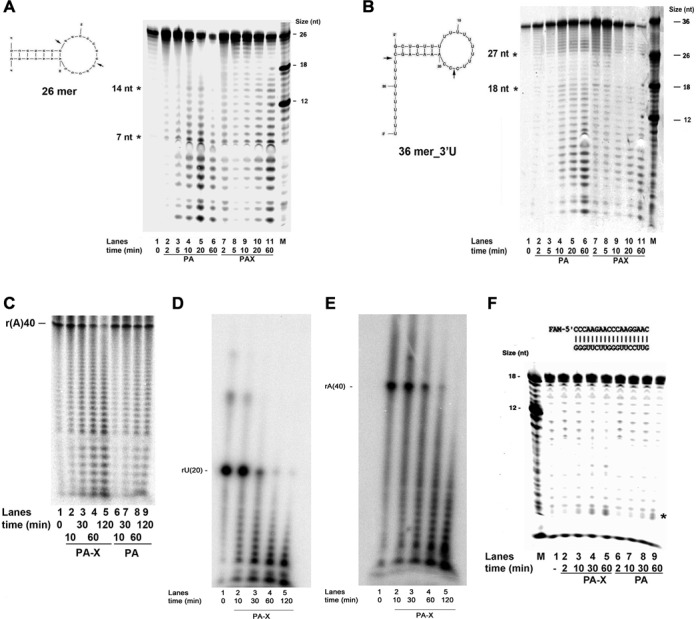
Comparison of PA and PA-X on different RNA substrates. The structure of the substrates used is shown next to the panels. M indicates the molecular size markers. (**A**) 2 pmols of PA (lanes 1–6) or PA-X (lanes 7–11) were tested on substrate 4 (Scheme 1). Asterisks mark the +7 and +14 cleavage products and arrows indicate their corresponding positions on the substrate sequence. (**B**) As in panel A, but in the presence of substrate 5. Asterisks mark the +18 and +27 cleavage products and arrows indicate their corresponding positions on the substrate sequence. (**C**) Comparison of endonuclease activities of 2 pmols of PA (lanes 1–4) or PA-X (lanes 6–9) on the ssRNA substrate 6 (Scheme 1). (**D**) Time course assay with 4 pmols of PA-X on substrate 11 (Scheme 1). (**E**) As in panel D, but in the presence of substrate 6. (**F**) As in panel C, but on the unstructured dsRNA substrate 7 (Scheme 1). The asterisk indicates the cleavage products.

In order to further investigate the different substrate specificities of PA and PA-X, a set of different RNA substrates was used (Scheme 1). First, substrate **4** was employed, which was predicted to form a single large terminal stem loop with a central ssRNA region extending from position +7 to +20. As shown in Figure 5A, already after 2 min, PA generated products ranging from +7 to +14 (lane 2), arising from the digestion within the ssRNA bubble. Only at later time points (lanes 3–6), products corresponding to the digestion of the 5′ end of the dsRNA stem appeared. On the other hand, PA-X generated a ladder of cleavage products all along the substrate, already at 2 min (lane 7). When a modified form of this substrate carrying a 10 nt poly-U tract at the 3′ end (substrate **5**, Scheme 1) was used, PA generated products > 27 nt, corresponding to the digestion of the 3′ end ssRNA tail (Figure 5B, lanes 2, 3), while at later time points, also products < 18 nt accumulated, corresponding to the digestion of the substrate starting from the ssRNA looped region (lanes 4–6). PA-X showed a similar pattern of cleavage products, but with a stronger accumulation of products at early time points with respect to PA (lanes 7–11). Quantification of the products as a function of time, allowed the calculation of the apparent rates of digestion. A summarized in Table 2, PA-X was 2.7-fold more active on substrate **5** than on substrate **4**, while the difference for PA was only 1.6-fold. Thus, the presence of long unstructured ssRNA seemed to stimulate PA-X activity. PA and PA-X were also compared on a poly r(A)40 ssRNA (substrate **6**). As shown in Figure 5C and Table 2, PA-X was 3.7-fold more active than PA on this substrate. To verify whether this reflected a sequence specificity, PA-X was additionally tested on a ss poly r(U)20 oligonucleotide (substrate **11**, Scheme 1) and on a 18mer ssRNA (18mer_p, Table 1). As shown in Figure 5D and E and summarized in Table 2, PA-X was slightly more active on the homopolymeric r(A)40 and r(U)20 ssRNA, with respect to the heteropolymeric 18mer, but did not show any sequence preference between poly r(A) and poly r(U). Finally, PA and PA-X were tested on fully dsRNA (substrate **7**, Scheme 1). As shown in Figure 5F and Table 2, PA was poorly active on this substrate, showing a 10-fold reduction of activity with respect to substrate 1, while PA-X retained significant cleavage activity (3-fold lower than on substrate 1). These data indicated differences in the catalytic activity between PA and PA-X.

### Mapping of the cleavage sites of PA and PA-X

**Figure 6.**
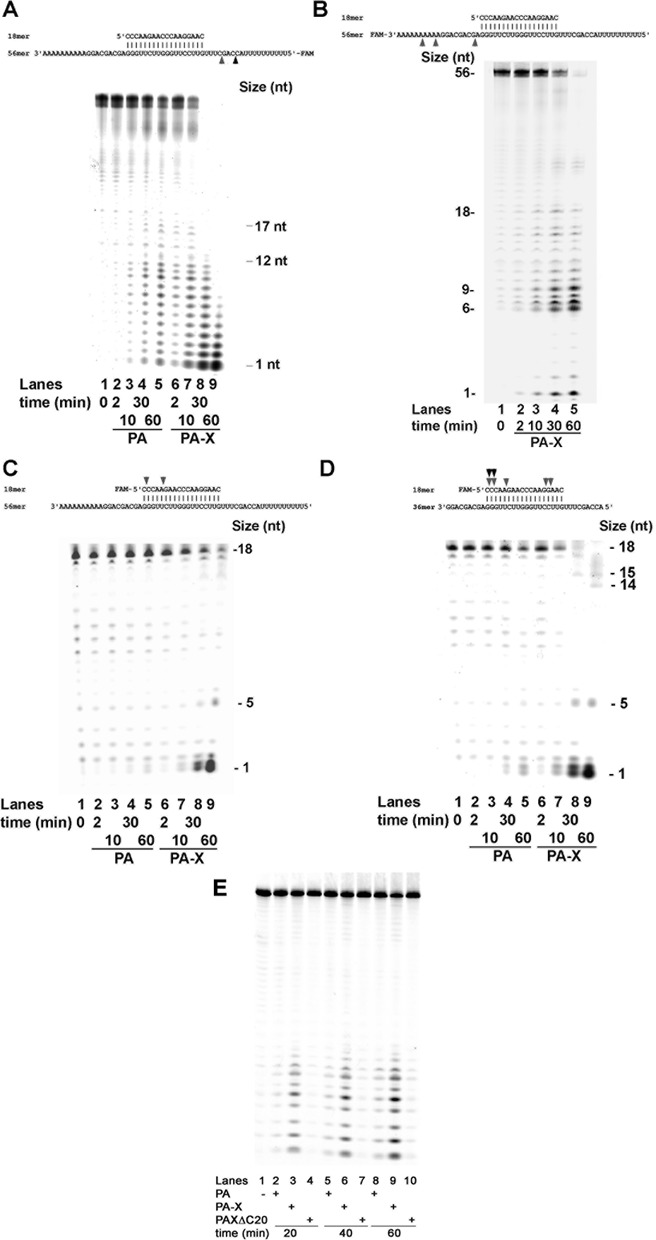
Influence of ssRNA regions on the endonuclease activity of PA and PA-X on linear RNA substrates. All substrate structures are shown on the top of the corresponding panels. Grey and black arrows indicate the cutting points of PA-X and PA, respectively. (**A**) PA (lanes 2–5) or PA-X (lanes 6–9) were tested on substrate 8 (Scheme 1), labelled at the 5′ end of the 56mer strand. M indicates molecular size markers. Lane 1, negative control in the absence of enzyme. (**B**) PA-X was tested on substrate 8 labelled at 3′ end of the 56mer strand. Lane 1, negative control in the absence of enzyme. (**C**) As in panel A, but with substrate 8 labelled at the 5′-end of 18mer strand. (**D**) PA (lanes 2–5) or PA-X (lanes 6–9), were tested on substrate 9 (Scheme 1). Lane 1, negative control in the absence of enzyme. (**E**) PA (lanes 2, 5, 8), PA-X (lanes 3, 6, 9) or PAXΔC20 (lanes 4, 7, 10) were tested on substrate 1 (Scheme 1). Lane 1, negative controls in the absence of enzyme.

In order to better understand the differences in cleavage specificity of PA and PA-X, a linear RNA 56mer substrate with a central 18 nt ds region (substrate **8**, Scheme 1) was employed. The 56mer strand was labelled at its 5′-end and the substrate was incubated with both PA and PA-X. Due to the presence of the partially ds region, this substrate was predicted not to form secondary structures. As shown in Figure 6A, PA-X proved to be more active than PA, leading to complete degradation of the substrate at late time points (Figure 6A, compare lanes 4 and 5 with lanes 8 and 9). At early time points, PA started to generate products from 12 nt to 1 nt (lanes 2 and 3), while PA-X already at 2 min generated slightly longer products of 17 nt (lane 6). Both cleavage patterns were consistent with the digestion occurring in the ssRNA region. In order to verify whether the cleavage was also occurring at the 3′ side, the label on substrate **8** was placed at the 3′ end. As shown in Figure 6B, when PA-X was incubated with this substrate, robust cleavage activity was observed, corresponding to the digestion of the 3′ ssRNA overhang. Thus, PA-X does not have a preferred directionality for RNA digestion, consistent with its activity as an endo- rather than an exo-nuclease.

To further explore the digestion pattern by PA and PA-X, the label on substrate **8** was placed also at the 5′ end of the short 18mer strand, thus allowing investigation of the cleavage of the dsRNA portion. As shown in Figure 6C, PA was completely inactive on this substrate, (Figure 6C, lanes 1–5). PA-X, on the other hand, was able to cleave also the dsRNA part, leading to almost complete digestion of the substrate (lanes 8 and 9). To understand whether the length of the ssRNA overhangs could influence the ability of PA and PA-X of cleaving inside the dsRNA portion, a shorter version of substrate **8** was used, without the 3′ poly (A) and 5′ poly (U) tails, thus bearing shorter ssRNA overhangs (substrate **9**, Scheme 1). As shown in Figure 6D, shortening the ssRNA overhangs resulted in an increased activity of PA-X (lanes 6–9) as well as of PA (lanes 2–5). However, also on this shorter substrate, PA-X was much more efficient in cleaving the dsRNA part than PA. Interestingly, the position of the first cleavage by PA on substrate **9** was of at position +1/+2 at the 5′ side of the dsRNA region. This corresponded to a distance of 11–12 nt from the 3′ end of the ss overhang (see Figure 6D, top scheme and lane 4). This was the size also consistently observed for the longest products generated (from the 5′ side) on substrate **8** (see Figure 6A, lane 2). Thus, it is possible to hypothesize that PA covers ≈11–12 nt of ssRNA, when the position to be cleaved is in the catalytic site. By shortening the 3′ ssRNA overhang on substrate **9**, the active site would have reached the beginning of the dsRNA part (Table 2), thus explaining the cleavage observed with the shorter substrate. PA-X seems to be less constrained by the size of the ssRNA region than PA, being able to cut further away from the substrate ends (Figure 6A, lane 2; Figure 6D, lane 8). Overall, these results confirmed differences between PA-X and PA.

### The natural deleted mutant PAXΔC20 has reduced endonucleolytic activity

Since PA, PA-X and PAXΔC20, all share the same catalytic domain, in order to understand whether their different C-ter portions may modulate the enzymatic activity, we compared their catalytic activities with the isolated PA catalytic domain (PAcd), representing the first 191 aa in common among all proteins. As shown in Figure 6E, on substrate **1** PAXΔC20 was significantly less active than PA or PA-X (for example compare lanes 4, 7, 10 with lanes 3, 6, 9). We next determined the kinetic parameters *K*_m_ and *V*_max_ and the catalytic efficiency *V*_max_/*K*_m_, for all four proteins. As reported in Table 3, PAXΔC20 on substrate **1** showed a 33-fold lower activity (*V*_max_/*K*_m_ value), with respect to PA-X full length and a 6.7-fold reduction with respect to PAcd. The PAcd itself showed lower activity with respect to PA full length (4.7-fold) and PA-X (3.3-fold). Similarly, on substrate **6**, the preferred substrate for PA-X, the catalytic efficiency of PAXΔC20 was 7-fold lower than the one of PA-X (Table 3). The reduced catalytic efficiency of PAXΔC20 with respect to PA-X was due to both an increased *K*_m_ for the RNA substrates (lower affinity) and a decreased *V*_max_. Collectively, these results suggest that the C-ter domain of PA and PA-X, albeit different, increased the catalytic activity of the enzyme with respect to the core N-terminal catalytic domain alone. Surprisingly, however, the natural 20 aa deletion present in PAXΔC20 showed a severe deficiency in its endonucleolytic activity.

**Table 3.** Kinetic parameters for RNA digestion by PA and PA-X proteins

	*K*_m_^a^ (μM)	*V*_max_ (pmols x min^−1^)	*V*_max_/*K*_m_
Substrate 1			
PA	3.3 ± 0.6	0.31 ± 0.03	0.094
PA-X	6.5 ± 0.3	0.43 ± 0.01	0.066
PAXΔC20	10 ± 2	0.037 ± 0.006	0.003
PAcd	5.5 ± 0.5	0.11 ± 0.007	0.02
Substrate 6			
PA	9.4 ± 0.9	0.29 ± 0.05	0.031
PA-X	4.2 ± 0.3	0.27 ± 0.01	0.064
PAXΔC20	13.4 ± 0.8	0.12 ± 0.006	0.009

^a^Kinetic parameters and the corresponding ±S.D. have been calculated as detailed in the Materials and Methods section.

## DISCUSSION

The endonucleolytic activity of the PA subunit of the RdRP of IAVs, removes the 5′-capped terminus of cellular pre-mRNAs, generating 5′-capped RNA oligonucleotides of 10–13 nt, which are used to prime the synthesis of viral mRNAs (7). Whether base-pairing between the vRNA template strand and the incoming 5′-capped leader pre-mRNA is required for cleavage is still unclear. Indeed, recent work provided compelling evidence that priming through base-pairing before cleavage is important, with the first 2–3 base paired residues playing a major role in stabilizing the cleavage complex (19,20). The base complementarity was found optimal when positioned 10–11 nt downstream the 5′ cap of the leader cellular RNA, consistent with the cap-snatched products generated *in vivo*.

The results presented here, lend further mechanistic support to these observations. In our study, PA was found to be able to preferentially cut ssRNA regions embedded in dsRNA molecules, such as bubbles or loops. These secondary structures are likely to be formed through imperfect matching between the leader RNA sequence and the vRNA template. In addition, when assessed on linear dsRNA templates bearing ssRNA overhangs, PA cleavage products were consistent with the enzyme cutting the ssRNA region about 10–12 nt downstream from the end of the RNA strand. This is consistent with the results mentioned above and may suggest that the size of the cleavage product corresponds to the size of the ssRNA region that is covered by the enzyme once bound to the substrate. Based on our findings, it is possible to hypothesize that, when the leader cellular RNA, upon binding to PB2 through its 5′ cap, is brought in contact with PA, the alignment between the leader RNA and the 3′ end of vRNA is stabilized through random base-pairing. Cleavage by PA may than occur in correspondence of ds-to-ssRNA junction, i.e. within bubbles formed by the presence of non-complementary regions between the leader RNA and the vRNA. The size of the cleaved product may be determined by the position of the PA active site, which is located 10–12 nt downstream the 5′ capped end. That the size of the cleaved products may depend upon the structural arrangement of the PA subunit, is also suggested by the observation that other cap-snatching viruses show a different cleavage pattern. For example, the Tomato Spotted Wilt Virus has preference for base pairing regions 16 nt downstream the 5′ cap (21). This hypothesis may be directly tested in future by *in vitro* transcription assays, using leader RNAs with different degrees of complementarity to the vRNA 3′ end.

Recently, it has been shown that a second protein, PA-X, is translated from the IAV genomic segment 3 encoding PA, through a ribosomal frameshifting event (11). Since PA and PA-X share the same N-terminal domain containing the endonuclease catalytic site, it has been hypothesized that PA-X could also be an endonuclease and indeed indirect evidence in transfection reporter systems, using a PA-X with mutations in the endonuclease active site, suggested that PA-X represses cellular gene expression through its endonucleolytic activity (11). Here, we have directly shown that recombinant purified PA-X has a robust endonucleolytic activity, which is capable of digesting both ss and dsRNA, showing a preference for ssRNA substrates. Interestingly, by comparing PA, PA-X and the isolated N-terminal catalytic domain, our data indicate that the different C-ter part is at least in part responsible for the differences in the catalytic activity between PA and PA-X. Overall, the enzymatic properties of PA and PA-X, as revealed in this study, are consistent with the proposed different roles played by these proteins in the IAV replicative cycle. PA seems to be very specific in cutting ssRNA when present in secondary structures such as loops and bubbles. As mentioned above, this ability would be important in facilitating the cap-snatching process. PA-X, on the other hand, seems to be more active on homopolymeric ssRNA, such as poly r(A) or poly r(U), but also capable of digesting dsRNA. Such characteristics make PA-X well suited to attack and destroy various host RNA species.

The PA-X protein is highly conserved among IAVs. However, a natural variant has been found in a subset of IAVs lineages, bearing a nonsense mutation at codon 42, resulting in a truncated PA-X protein lacking the last 20 aa (11,13). This PAXΔC20 protein variant is present in IAVs infecting dogs (H3N2 and H3N8) and swine (H1N1), and in the human pandemic H1N1/2009 strains, which acquired this mutation from swine viruses (13). Phylogenetic analysis revealed that this truncated version has evolved independently four times from a full length PA-X gene: twice in swine and twice in dogs (13). Evidence for a selective pressure leading to the fixation of the PAXΔC20 variant in particular host species (swine and dogs) has been taken as an indication that the mutant protein has distinct functional properties with respect to the full length PA, which may be correlated to the adaptation of IAVs to these new hosts. However, such hypothesis has not been experimentally confirmed yet.

In this work, we provide, to the best of our knowledge for the first time, direct evidence that the recombinant PAXΔC20 variant protein has severely reduced enzymatic activity, when compared to PA-X and PA. Our results suggest that the last 20 aa at the C-ter of PA-X, which are not part of the active site, are important for the activity. Crystal structures of PA, both alone or in complex with the other subunits and RNA, have shown that the PA C-ter region (aa 631–716) folds into three α-helices, which closely interact to form part of the PB1 binding site (8,22–23). In addition, interaction of full length PA and RNA has been proposed to involve Arg196 located on the α7 helix in the C-ter domain (24). Since PA-X must necessarily adopt a more compact conformation than PA, owing to its smaller size, it is possible that its C-ter part may fulfil the same role as in PA, modulating RNA binding. Preliminary RNA binding measurements done in our laboratory seem to support this hypothesis (data not shown), that, however, must be assessed through the determination of the crystal structure of PA-X in complex with ssRNA.

In a previous work, the effects of PA-X in modulating gene expression have been compared using an A/Brevig Mission/H1N1/1918 genetic background. The authors showed that lack of PA-X expression increased the overall pathogenicity of H1N1/1918 in mice, leading to differential expression (both up- and down-regulation) of > 5000 genes (11). Two recent reports (14,15) confirmed these preliminary findings, showing that loss of PA-X increased the pathogenicity of the human pandemic H1N1/2009 and avian influenza H5N1 IAV strains. Recently, Oishi et al. (25), creating a series of C-ter deletion mutants forms of PA-X, demonstrated that the first 15 aa of the C-ter domain of PA-X are sufficient for full shutoff activity, while they did not observe a strong contribution of the last 20 aa. However, another recent study (26), analysed the effects of the variant PA-X form, bearing the C-ter 20 aa deletion. The authors discovered that IAV belonging to three different strains (human pandemic H1N1 2009, avian H5N1 and avian H9N2), consistently showed reduced viral replication and pathogenicity when expressing the truncated PA-X protein lacking the last 20 aa. In addition, they showed that the last 20 C-ter aa of PA-X were directly implicated in the shutoff of host protein synthesis. Given that host shutoff has been ascribed to the endonucleolytic activity of PA-X (11–12,27), these data may suggest that the C-ter domain of PA-X may play a role in modulating its enzymatic activity. These data are in agreement with our observation that the truncation at codon 42 of PA-X, as recapitulated in the PAXΔC20 mutant, severely reduced its enzymatic activity. These observations, combined with the evidence for positive selection of this mutation (13), may suggest that reduced PA-X activity may also be advantageous to the virus. Our PA, PA-X and PAXΔC20 proteins are derived from the low pathogenic human A/WSN/33 (H1N1) strain. It has been shown that pandemic H1N1 2009 strains carrying a deleted PA-X had stronger shutoff activity than the A/WSN/33 (H1N1) virus carrying a full length PA-X (26). Thus, whether the role of the C-ter domain of PA-X shown here will be recapitulated in other viral strains awaits further experimental confirmation. It is interesting, however, that Desmet at al. (27) observed a stronger shutoff activity by A/WSN/33 PA-X, with respect to the PA N-ter domain (aa 1–186) containing the endonuclease active site. We also observed a substantially lower endonuclease activity of the isolated PA catalytic domain (aa 1–191), with respect to full length PA-X.

Due to its essential role in viral replication, the endonuclease activity of PA is being considered as an attractive target for antiviral chemotherapy. Here we show that both PA and PA-X are inhibited by the small molecule DBPA. Besides demonstrating the possibility of pharmacologically modulating PA-X activity, these results also indicate that it would be important to include PA-X testing in the drug development process, when attempting to develop selective PA inhibitors.

## Supplementary Material

SUPPLEMENTARY DATA

## References

[1] Bavagnoli L., Maga G. (2011). The 2009 influenza pandemic: promising lessons for antiviral therapy for future outbreaks. Curr. Med. Chem..

[2] Wise H.M., Hutchinson E.C., Jagger B.W., Stuart A.D., Kang Z.H., Robb N., Schwartzman L.M., Kash J.C., Fodor E., Firth A.E. (2012). Identification of a novel splice variant form of the influenza A virus M2 ion channel with an antigenically distinct ectodomain. PLoS Pathog..

[3] Muramoto Y., Noda T., Kawakami E., Akkina R., Kawaoka Y. (2013). Identification of novel influenza A virus proteins translated from PA mRNA. J. Virol..

[4] Datta K., Wolkerstorfer A., Szolar O.H., Cusack S., Klumpp K. (2013). Characterization of PA-N terminal domain of Influenza A polymerase reveals sequence specific RNA cleavage. Nucleic Acids Res..

[5] Plotch S.J., Bouloy M., Ulmanen I., Krug R.M. (1981). A unique cap(m7GpppXm)-dependent influenza virion endonuclease cleaves capped RNAs to generate the primers that initiate viral RNA transcription. Cell.

[6] Ruigrok R.W., Crepin T., Hart D.J., Cusack S. (2010). Towards an atomic resolution understanding of the influenza virus replication machinery. Curr. Opin. Struct. Biol..

[7] Dias A., Bouvier D., Crepin T., McCarthy A.A., Hart D.J., Baudin F., Cusack S., Ruigrok R.W. (2009). The cap-snatching endonuclease of influenza virus polymerase resides in the PA subunit. Nature.

[8] Yuan P., Bartlam M., Lou Z., Chen S., Zhou J., He X., Lv Z., Ge R., Li X., Deng T. (2009). Crystal structure of an avian influenza polymerase PA(N) reveals an endonuclease active site. Nature.

[9] Noble E., Cox A., Deval J., Kim B. (2012). Endonuclease substrate selectivity characterized with full-length PA of influenza A virus polymerase. Virology.

[10] Crepin T., Dias A., Palencia A., Swale C., Cusack S., Ruigrok R.W. (2010). Mutational and metal binding analysis of the endonuclease domain of the influenza virus polymerase PA subunit. J. Virol..

[11] Jagger B.W., Wise H.M., Kash J.C., Walters K.A., Wills N.M., Xiao Y.L., Dunfee R.L., Schwartzman L.M., Ozinsky A., Bell G.L. (2012). An overlapping protein-coding region in influenza A virus segment 3 modulates the host response. Science.

[12] Hayashi T., MacDonald L.A., Takimoto T. (2015). Influenza A Virus Protein PA-X Contributes to Viral Growth and Suppression of the Host Antiviral and Immune Responses. J. Virol..

[13] Shi M., Jagger B.W., Wise H.M., Digard P., Holmes E.C., Taubenberger J.K. (2012). Evolutionary conservation of the PA-X open reading frame in segment 3 of influenza A virus. J. Virol..

[14] Hu J., Mo Y., Wang X., Gu M., Hu Z., Zhong L., Wu Q., Hao X., Hu S., Liu W. (2015). PA-X Decreases the Pathogenicity of Highly Pathogenic H5N1 Influenza A Virus in Avian Species by Inhibiting Virus replication and Host Response. J. Virol..

[15] Gao H., Sun Y., Hu J., Qi L., Wang J., Xiong X., Wang Y., He Q., Lin Y., Kong W. (2015). The contribution of PA-X to the virulence of pandemic 2009 H1N1 and highly pathogenic H5N1 avian influenza viruses. Sci. Rep..

[16] Rash A., Woodward A., Bryant N., McCauley J., Elton D. (2014). An efficient genome sequencing method for equine influenza [H3N8] virus reveals a new polymorphism in the PA-X protein. Virol. J..

[17] Tomassini J., Selnick H., Davies M.E., Armstrong M.E., Baldwin J., Bourgeois M., Hastings J., Hazuda D., Lewis J., McClements W. (1994). Inhibition of cap (m7GpppXm)-dependent endonuclease of influenza virus by 4-substituted 2,4-dioxobutanoic acid compounds. Antimicrob. Agents Chemother..

[18] Zuker M. (2003). Mfold web server for nucleic acid folding and hybridization prediction. Nucleic Acids Res..

[19] Geerts-Dimitriadou C., Zwart M.P., Goldbach R., Kormelink R. (2011). Base-pairing promotes leader selection to prime in vitro influenza genome transcription. Virology.

[20] Geerts-Dimitriadou C., Goldbach R., Kormelink R. (2011). Preferential use of RNA leader sequences during influenza A transcription initiation in vivo. Virology.

[21] van Knippenberg I., Lamine M., Goldbach R., Kormelink R. (2005). Tomato spotted wilt virus transcriptase in vitro displays a preference for cap donors with multiple base complementarity to the viral template. Virology.

[22] Obayashi E., Yoshida H., Kawai F., Shibayama N., Kawaguchi A., Nagata K., Tame J.R., Park S.Y. (2008). The structural basis for an essential subunit interaction in influenza virus RNA polymerase. Nature.

[23] Pflug A., Guilligay D., Reich S., Cusack S. (2014). Structure of influenza A polymerase bound to the viral RNA promoter. Nature.

[24] Xiao S., Klein M.L., LeBard D.N., Levine B.G., Liang H., MacDermaid C.M., Alfonso-Prieto M. (2014). Magnesium-dependent RNA binding to the PA endonuclease domain of the avian influenza polymerase. J. Phys. Chem. B.

[25] Oishi K., Yamayoshi S., Kawaoka Y. (2015). Mapping of a Region of the PA-X Protein of Influenza A Virus That Is Important for Its Shutoff Activity. J. Virol..

[26] Gao H., Sun H., Hu J., Qi L., Wang J., Xiong X., Wang Y., He Q., Lin Y., Kong W. (2015). The 20 amino acids at the C-terminus of PA-X are associated with increased influenza A virus replication and pathogenicity. J. Gen. Virol..

[27] Desmet E.A., Bussey K.A., Stone R., Takimoto T. (2013). Identification of the N-terminal domain of the influenza virus PA responsible for the suppression of host protein synthesis. J. Virol..

