# Supplementary material for: The novel influenza A virus protein PA-X and its naturally deleted variant show different enzymatic properties in comparison to the viral endonuclease PA
Source: Nucleic Acids Res. 2015 Sep 17;43(19):9405–17. doi: 10.1093/nar/gkv926 (PMC4627086; doi:10.1093/nar/gkv926)
Supplement: SUPPLEMENTARY DATA [file supp_43_19_9405__index.html]

The novel influenza A virus protein PA-X and its naturally deleted variant show different enzymatic properties in comparison to the viral endonuclease PA — The novel influenza A virus protein PA-X and its naturally deleted variant show different enzymatic properties in comparison to the viral endonuclease PA — SUPPLEMENTARY DATA 

# The novel influenza A virus protein PA-X and its naturally deleted variant show different enzymatic properties in comparison to the viral endonuclease PA

## SUPPLEMENTARY DATA

- SUPPLEMENTARY DATA
