# Supplementary material for: The novel influenza A virus protein PA-X and its naturally deleted variant show different enzymatic properties in comparison to the viral endonuclease PA
Source: Nucleic Acids Res. 2015 Sep 17;43(19):9405–17. doi: 10.1093/nar/gkv926 (PMC4627086; doi:10.1093/nar/gkv926)
Supplement: SUPPLEMENTARY DATA [file supp_gkv926_nar-00682-h-2015-File009.pdf]

Supplementary Information to the manuscript:

The novel influenza A virus protein PA-X and its naturally deleted variant show different enzymatic properties in comparison to the viral endonuclease PA.

Laura Bavagnoli[1], Stefano Cucuzza[1], Giulia Campanini[2], Francesca Rovida[2], Stefania Paolucci[2], Fausto Baldanti[2] and Giovanni Maga[1]*

1. Institute of Molecular Genetics IGM-CNR, via Abbiategrasso 207, 27100 Pavia (Italy)
2. Molecular Virology Unit, Microbiology and Virology Department, Fondazione IRCCS Policlinico San Matteo, P.le Golgi 2, 27100 Pavia, Italy.

Running title: Enzymatic characterization of influenza PA-X protein

Keywords: Influenza virus; RNA endonuclease; viral replication; enzyme kinetics

* Corresponding Author: Dr. Giovanni Maga, E-mail: maga@igm.cnr.it; Tel.: +390382546354; Fax: +390382422286.

Contents:

- Supplementary Figure Legends

- Supplementary Figures S1 – S3.

Supplementary Figure Legends

Supplementary Figure S1. Mock purification through a NiNTA column, starting from E.coli cells transformed with the empty pTrcHisA expression vector. Cells were grown, induced and lysed as described in the Methods section. The extract was applied to a NiNTA FPLC column. **A**. Elution profile of the NiNTA column, showing the protein peaks (absorbance units), the eluted volume, the fraction numbers and the linear Imidazole gradient. **B**. Coomassie staining of the SDS-PAGE of the purification. L, loading; FT, flow-through; W, wash. MWM, molecular weight markers. Fractions 5 to 10, corresponding to Imidazole concentrations from 150 mM to 375 mM were loaded on the gel. The typical elution point of PA is between 200 mM and 250 mM Imidazole. **C**. Western blot with anti-his antibodies of the same fractions shown in panel B. **D**. Endonuclease assay performed with fractions 4 to 11 (lanes 3 - 10) from the NiNTA column, incubated in the presence of Substrate **1**. Lane 1, positive control in the presence of 1 pmol of PA. Lane 2, negative control in the absence of enzyme.

Supplementary Figure S2. Determination of the kinetic parameters for PA-X activity on Substrate 1. **A**. Time course experiments with 2 pmols of PA-X in the presence of increasing amounts of Substrate **1**. Values are the means of two replicates. Error bars are ±S.D. Linear fitting was performed with the program GraphPad Prism. **B**. Plot of the slopes derived from the experiment shown in panel A (k_{app} values) as a function of the Substrate 1 concentrations. Data were fitted to Eq. (1) with the program GraphPad Prism (see Material and Methods).

Supplementary Figure S3. Sensitivity of PA and PA-X to the inhibitor DBPA. **A**. Substrate **1** was incubated in the absence (lane 1) or in the presence of PA alone (lane 2) or in combination with 1 mM DBPA. **B**. Substrate **1** was incubated in the absence (lane 1), or in the presence of PA alone (lane 2), in combination with DMSO alone (lane 3) or with increasing amounts of the inhibitor DBPA dissolved in DMSO (lanes 4 - 7). Final DMSO concentration in lanes 3 - 7 was 10%.

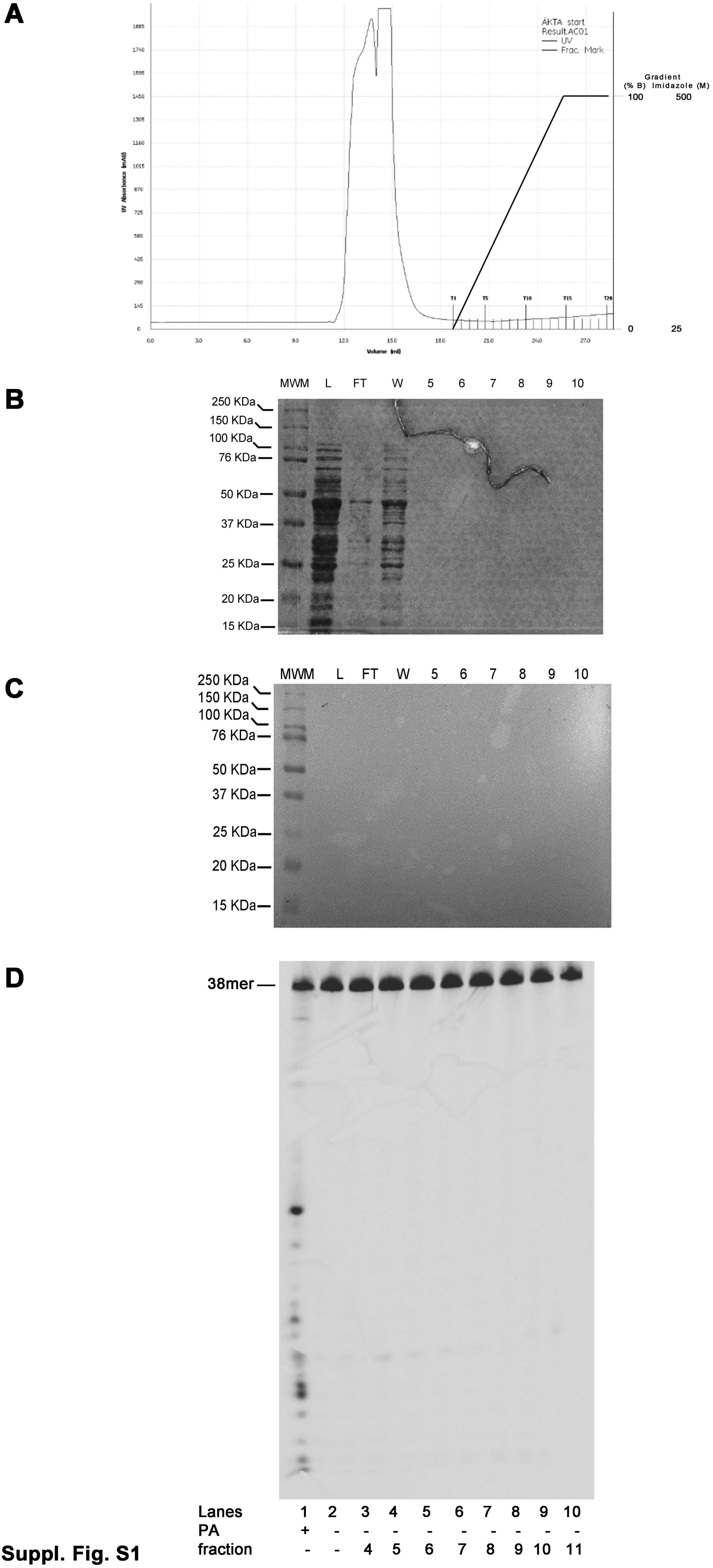
A
ÄKTA start
Result:AC01
UV
Frac. Mark
Gradient
(% B) Imidazole (M)
100
500
0
25
UV Absorbance (mAU)
Volume (ml)
T1
T5
T10
T15
T20
B
MWM L FT W 5 6 7 8 9 10
250 KDa
150 KDa
100 KDa
76 KDa
50 KDa
37 KDa
25 KDa
20 KDa
15 KDa
C
MWM L FT W 5 6 7 8 9 10
250 KDa
150 KDa
100 KDa
76 KDa
50 KDa
37 KDa
25 KDa
20 KDa
15 KDa
D
38mer
Lanes 1 2 3 4 5 6 7 8 9 10
PA + - - - - - - - - -
fraction - - 4 5 6 7 8 9 10 11

Suppl. Fig. S1

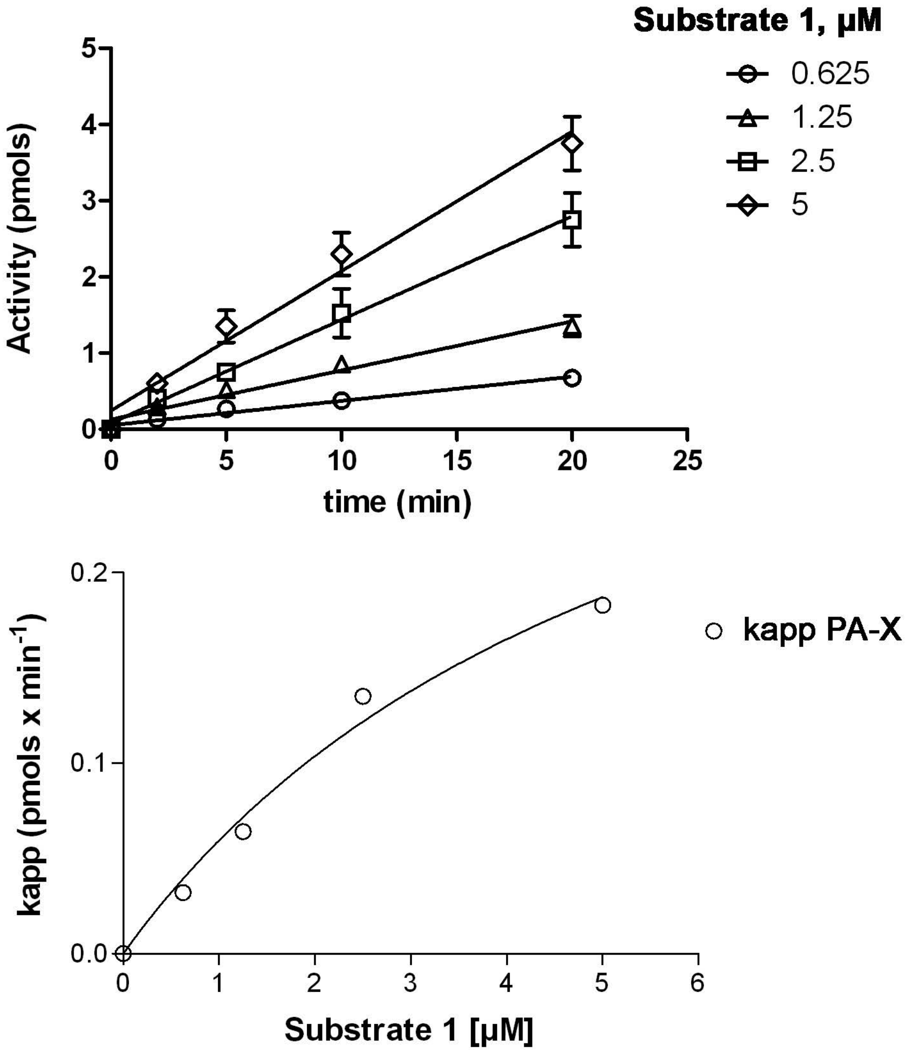
Substrate 1, µM
0.625
1.25
2.5
5
Activity (pmols)
time (min)
kapp (pmols x min-1)
kapp PA-X
Substrate 1 [µM]

Suppl. Fig. S2

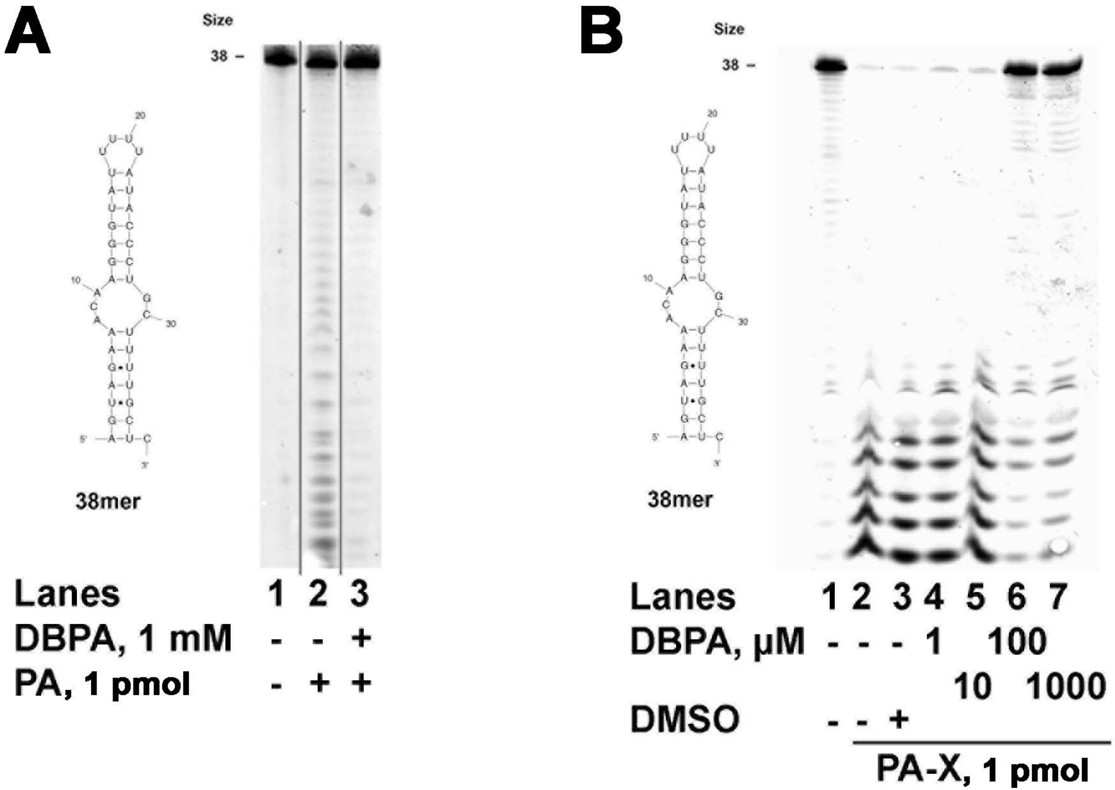
A
Size
38 –
38mer
Lanes 1 2 3
DBPA, 1 mM - - +
PA, 1 pmol - + +
B
Size
38 –
38mer
Lanes 1 2 3 4 5 6 7
DBPA, µM - - - 1 100
10 1000
DMSO - - +
PA-X, 1 pmol

Suppl. Fig. S3
